# A Randomized Controlled Trial Assessing the Release of Circulating Tumor and Mesenchymal Cells in No-Touch Radical Nephrectomy

**DOI:** 10.3390/cancers16213601

**Published:** 2024-10-25

**Authors:** Tito Palmela Leitão, Patrícia Corredeira, Carolina Rodrigues, Paulina Piairo, Miguel Miranda, Ana Cavaco, Sandra Kucharczak, Marília Antunes, Sara Peixoto, José Palma dos Reis, Tomé Lopes, Lorena Diéguez, Luís Costa

**Affiliations:** 1 Instituto de Medicina Molecular João Lobo Antunes, Faculdade de Medicina, Universidade de Lisboa, 1649-028 Lisboa, Portugal; pcorredeira@medicina.ulisboa.pt (P.C.); carolina.rodrigues@inl.int (C.R.); acmcavaco@ualg.pt (A.C.); sandraku@stud.ntnu.no (S.K.); luis.costa@chln.min-saude.pt (L.C.); 2 Faculdade de Medicina, Universidade de Lisboa, 1649-028 Lisboa, Portugal; 3 Urology Department, Hospital de Santa Maria, Centro Hospitalar Universitário Lisboa Norte, ULS Santa Maria, 1649-028 Lisboa, Portugal; miguel.soares.miranda@hospitaldaluz.pt; 4 International Iberian Nanotechnology Laboratory, 4715-330 Braga, Portugal; paulina.piairo@inl.int (P.P.); lorena.dieguez@inl.int (L.D.); 5 RUBYnanomed Lda, 4700-314 Braga, Portugal; 6 Department of Clinical and Molecular Medicine, Faculty of Medicine and Health Sciences, Norwegian University of Science and Technology, P.O. Box 8905, 7491 Trondheim, Norway; 7 CEAUL—Centro de Estatística e Aplicações, Faculdade de Ciências, Universidade de Lisboa, 1749-028 Lisboa, Portugal; marilia.antunes@ciencias.ulisboa.pt; 8 Radiology Department, Hospital de Santa Maria, Centro Hospitalar Universitário Lisboa Norte, ULS Santa Maria, 1649-028 Lisboa, Portugal; sara.peixoto@chln.min-saude.pt; 9 Oncology Department, Hospital de Santa Maria, Centro Hospitalar Universitário Lisboa Norte, ULS Santa Maria, 1649-028 Lisboa, Portugal

**Keywords:** circulating mesenchymal cell, circulating tumor cell, kidney cancer, laparoscopy, liquid biopsy, microfluidics, no-touch surgery, radical nephrectomy, renal cell carcinoma

## Abstract

Background: Circulating tumor cells (CTCs) may be the missing renal cell carcinoma (RCC) biomarker. No-touch (NT) resection has shown benefit in several tumors. Methods: A randomized controlled trial comparing CTC and circulating mesenchymal cell (CMC) release in no-touch (NT) vs. conventional (C) laparoscopic RN. Blood samples were collected at operation room arrival (S0), specimen extraction (S1), postoperative D1, and D30. CTCs were isolated and analyzed using RUBYchip™. Results: Thirty-four patients were included. No significant differences were found between groups in CTC and CMC counts, count variations between time points, complications, and survival. The total circulating cell detection rates in the NT, C, and overall RCC groups were 58.3%, 80.0%, and 70.4% at S0; 41.6%, 86.7%, and 66.7% at S1; 50.0%, 64.3%, and 60.0% at D1; and 54.5%, 42.9%, and 44.0% at D30, respectively. A progressive decrease in CMCs was observed in the C group after surgery, especially at D1 (4.78 to 1.64 CMCs/7.5 mL blood, *p* = 0.035). Healthy controls had no circulating cells; however, high CMC counts were found in chronic inflammation controls and oncocytoma patients, with no significant difference from RCC patients (*p* = 0.460). Conclusions: NT RN did not reduce circulating cell release nor improve survival compared to C RN.

## Simple Summary

This study compared a no-touch technique with conventional radical nephrectomy for the treatment of renal cell carcinoma in terms of circulating tumor cell (CTC) and circulating mesenchymal cell (CMC) release and patient prognosis. The results showed that the no-touch approach did not reduce CTC or CMC release or improve survival, suggesting that early pedicle ligation, the last standing Robson principle of radical nephrectomy, may have fallen. Nonetheless, the no-touch technique proved to be faster and as safe as the conventional radical nephrectomy. Healthy controls had no circulating cells; however, high CMC counts were found in chronic inflammation controls and oncocytoma patients, with no difference to RCC patients.

## 1. Introduction

Renal cell carcinoma (RCC) had a global incidence of 431,288 in 2020 [1]. Surgery is the preferred treatment for localized disease, although recurrence occurs in 20–40% of the cases [2]. Radical nephrectomy (RN) is performed in half of the cases whenever a partial nephrectomy is not feasible [3]. Despite a significant increase in early detection in recent years, one-third of the RCC patients still present with metastatic disease [1,4].

Circulating tumor cells (CTCs) have been described as a potential biomarker for RCC [5]. No clinically validated RCC biomarker is currently available. CTCs have been shown to correlate with RCC staging and survival [6,7,8]. The epithelial cell-adhesion molecule (EpCAM)-based CellSearch^®^ was the first FDA-approved platform for CTC analysis, providing prognostic information in metastatic breast, prostate, and colorectal cancer [9]. However, the fact that only 18.6% of RCCs express EpCAM raised the need for alternative approaches for CTC analysis in RCCs [6]. Maertens and colleagues reported that a cell size-based system was the most efficient CTC isolation platform in RCC cell lines [10].

Exploratory studies of perioperative CTC kinetics in RCC have shown an increase in CTC counts on day (D1) and a decrease on subsequent days [11,12,13,14]. Bluemke et al. found that preoperative CTC detection was associated with a relative risk of death of 2.7 (*p* = 0.049) and postoperative detection with a relative risk of death of 4.3 (*p* = 0.036) [13]. These results suggested that intraoperative tumor manipulation increases CTC release in RCC surgery and impacts prognosis.

The concept of no-touch (NT) tumor resection was first introduced in 1977 in colorectal cancer [15]. In one study, fewer tumor mutations were detected by RT-PCR in patients undergoing NT compared to conventional (C) resection (73% vs. 14%; *p* = 0.05) [16]. However, two randomized control trials (RCTs) failed to demonstrate a difference in outcomes in favor of NT resection in colorectal cancer [17,18]. In a prospective lung cancer trial, CTC detection after surgery was significantly lower in the NT compared to the C approach (12.5 vs. 85.7%; *p* = 0.02) [19].

RN has been classically performed according to the five Robson principles of 1963 [20]. All of them have been refuted except for early renal pedicle ligation. A few publications have described this technique as safe, although with no benefit in outcomes [21,22,23]. To the best of our knowledge, there are no publications addressing the NT technique for RN.

The objective of this study was to investigate whether the surgical manipulation of the kidney during RN increases circulating cell (CC) release and whether CC release can be reduced by using an NT technique. The associations between CC kinetics and patient prognosis and R0 status were secondary objectives.

## 2. Materials and Methods

### 2.1. Study Design and Participants

This study was a prospective RCT conducted at the Department of Urology of Centro Hospitalar Universitário Lisboa Norte (CHLUN) between September 2021 and April 2022 (Supplementary Figure S1). Patients with a renal mass > 4 cm with an indication for laparoscopic RN (LRN) were included. The exclusion criteria included patients with a history of other cancers, age < 18 years, pregnant, with contraindications for laparoscopic surgery (peritoneal dialysis patients), or surgically unfit. All the patients provided written informed consent to participate in the study. This study was designed in accordance with the CONSORT 2010 Statement (Figures S4 and S5).

Two groups were compared: an NT LRN (NT) group and a C LRN (C) group.

The patients were positioned in lateral decubitus. Two 12 mm ports were placed at the pararectal and midclavicular lines, and two 5 mm ports were placed at the midaxillary line and 2 cm from the anterior superior iliac spine. An optional 5 mm port was placed for liver retraction. The colon was retracted along the avascular plane just anterior to Gerota’s fascia until the vena cava on the right and the aorta on the left were exposed. The second part of the duodenum was reflected medially on the right side.

Thereafter, the surgical protocol for the NT group was to incise Gerota’s fascia just above the renal vein and to immediately and selectively ligate the renal pedicle using Hem-o-Lok^®^ clips. No kidney manipulation was performed until this point. The surgery then proceeded in the usual way.

In group C, the surgical protocol consisted of the identification and superior retraction of the ureter together with the lower pole of the kidney, while the dissection continued cephalad until the renal pedicle was reached. The latter was then selectively ligated as previously described while maintaining renal traction.

Patients presenting with an indication for total nephrectomy due to hypofunctioning kidneys and without a history of cancer were used as controls and divided into two subgroups: (1) patients with systemic inflammation (inflammatory controls), defined by severe/recurrent pyelonephritis/pyonephrosis, elevated serum inflammatory parameters, and chronic pyelonephritis on pathology; and (2) patients with atrophic kidneys and none of the above criteria (healthy controls).

### 2.2. Randomization and Masking

The patients were randomly assigned to either group using a computer-generated allocation sequence. The allocation was disclosed to the surgical team upon the patient’s arrival in the operating room (OR).

### 2.3. Blood Collection and CTC Isolation and Characterization

A 7.5 mL peripheral venous blood sample was collected in EDTA tubes on arrival at the OR (S0), after specimen extraction (S1), and on postoperative D1 and D30. A single blood sample was collected from the study controls on the day of enrollment.

The anonymized samples were processed in the RUBYchip™ microfluidic device at 80 μL/min, as previously described [24]. CCs were identified via immunocytochemistry using AF647-conjugated anti-human vimentin (Vim; Biolegend (San Diego, CA, USA), 1:50), PE-conjugated anti-human CD45 (Invitrogen, Thermo Fisher Scientific (Waltham, MA, USA), 1:50), FITC-conjugated anti-human cytokeratin (CK), and DAPI (1 μg/mL). Fluorescent images were captured with an Allegro Plus microscope (BioView, Rehovot, Israel) at 20× magnification.

CCs were identified by morphology (membrane integrity and round nucleus) and phenotype (DAPI+/CD45−/CK+ for epithelial CTCs, and DAPI+/CD45−/CK+/Vim+ for epithelial–mesenchymal transition CTCs). Circulating mesenchymal cells (CMCs) were DAPI+/CD45−/CK−/Vim+, and clusters were groups of at least two cells with these traits.

### 2.4. Outcomes and Statistical Analysis

A sample size of 34 patients was calculated to detect a 20% decrease in CTCn variation after surgery in the NT group, assuming a Poisson distribution. The Kruskal–Wallis test was used to assess group homogeneity, followed by pairwise comparisons (Dunn’s test). The Wilcoxon signed-rank test was used to compare CC counts between the groups and time points. The Mann–Whitney test was used to compare the absolute cell count differences between the groups. The Wilcoxon rank sum test was used to compare the relative cell count differences between the groups. Spearman’s correlation analysis was performed between the cell counts and quantitative clinical and imaging variables. Kaplan–Meier was used for progression-free (PFS) and overall survival (OS) analyses. Bonferroni corrections were used for multiple hypothesis testing. A *p*-value ≥ 0.05 was considered statistically significant. Statistical analyses were performed using the R software v2022.07.1 (R Foundation for Statistical Computing, Vienna, Austria).

## 3. Results

**Figure 1:**
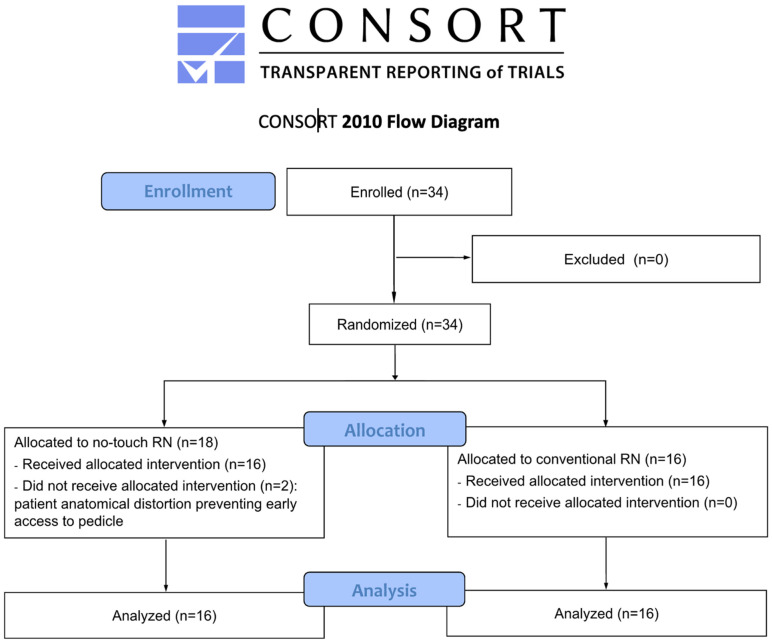
CONSORT flow diagram.

Thirty-four patients were randomly assigned to the NT (n = 18) and C (n = 16) groups (Figure 1).

The baseline clinicopathological characteristics of the study participants and controls are shown in Table 1.

The groups were balanced for all the demographic and clinicopathologic characteristics. The operative time was significantly shorter in the NT group compared to the C group (84.5 min [min], interquartile range [IQR] 31.5 vs. 107.5 min, IQR 35.5; *p* = 0.015), as was the time to renal vein exposure (21.0 min, IQR 10.5 vs. 37.0 min, IQR 26.7 min; *p* < 0.001).

Five patients had non-malignant histology: oncocytoma in four and focal xanthogranulomatous pyelonephritis (XP) in one. Only RCC patients were included in the intervention groups for CTC and CMC counts.

**Figure 2:**
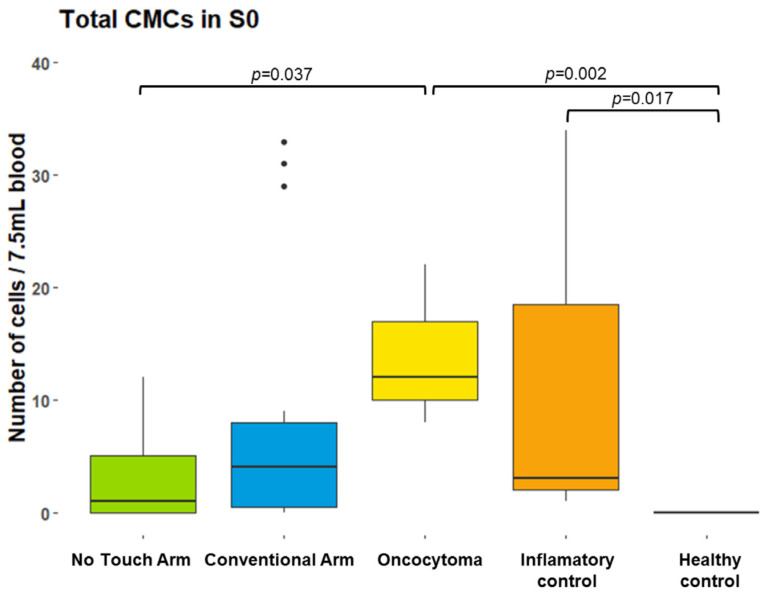
Box plots comparing the total CMC counts at S0 between the NT group, C group, oncocytoma patients, inflammatory controls, and healthy controls. CMC, circulating mesenchymal cell; NT, no-touch; S0, time of arrival in the operating room (baseline).

The baseline (S0) CC counts in the intervention and control groups are shown in Table 2 and Figure 2.

No CTCs were detected in the control groups or in the oncocytoma patients. No CMCs were detected in the healthy control group (Figure 2 and Figure S2). However, all the patients in the chronic inflammation control group had a significantly higher number of CMCs compared to the healthy controls (mean 22.5 [range 1–52] cells per 7.5 mL of blood (*p* = 0.007), but no significant count differences compared to the RCC NT and C groups (*p* = 0.291 and *p* = 0.460, respectively). CMCs were also detected in the oncocytoma patients at significantly higher counts compared to the RCC NT group (*p* = 0.037), but with no count differences compared to the RCC C group (*p* = 1.000) or the chronic inflammation control group (*p* = 0.205).

No correlation was found between the CMC counts and serum inflammatory parameters, namely C-reactive protein and leukocyte and neutrophil counts (Table S4).

Table 3 shows the CC counts and characteristics in each intervention group at each considered time point.

The total CC detection rates in the NT, C, and RCC groups were 58.3%, 80.0%, and 70.4% at S0; 41.6%, 86.7%, and 66.7% at S1; 50.0%, 64.3%, and 60.0% at D1; and 54.5%, 42.9%, and 44.0% at D30, respectively (Table S3.1). No differences on CC count between clear cell and non-clear cell RCC were found (Table S3.3).

The CTC detection rates in the NT, C, and RCC groups were 0%, 6.7%, and 3.7% at both S0 and S1; 27.3%, 7.1%, and 16.0% at D1; and 9.1%, 14.3%, and 12.0% at D30, respectively.

Most CCs were CMCs in all the groups and time points. Single CMCs were found at all the time points and in all the groups in one patient with focal XP at S1. CMC clusters were found in all the groups at all the time points except D1, and in the NT and XP groups at S1.

The CMC detection rates in the RCC NT, RCC C, and RCC groups were 58.3%, 73.3%, and 81.5% at S0; 41.7%, 80.0%, and 63.0% at S1; 36.4%, 57.1%, and 48.0% at D1; and 45.5%, 42.9%, and 44.0% at D30, respectively.

CMC clusters were found in 16.7%, 20.0%, and 18.5% of the patients in the NT, C, and whole RCC groups at S0; 0%, 6.7%, and 3.7% at S1; 0%, 0%, and 0% at D1; and 13.3%, 0.0%, and 7.4% at D30.

**Figure 3:**
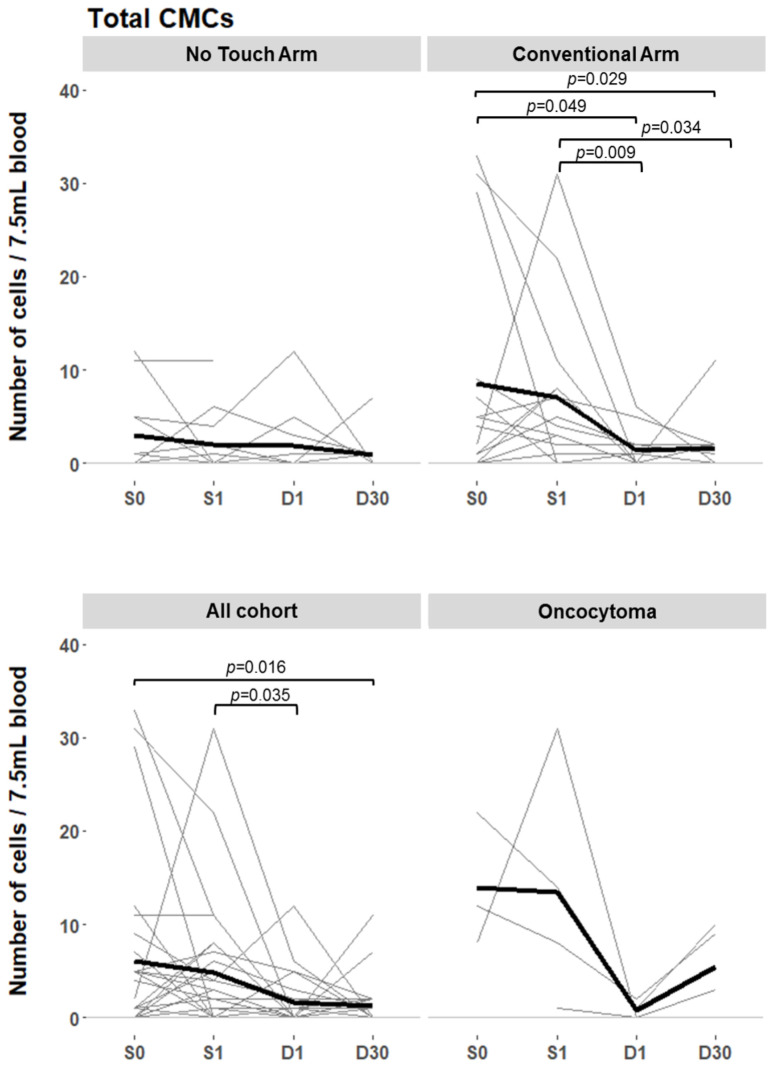
CMC counts at different time points (S0, S1, D1, and D30) for the NT and C groups (**top figure**) and for the total cohort and oncocytoma groups (**bottom figure**). Individual patients are shown as gray lines and the mean as a black line. S0, blood sample collected on arrival in the operating room; S1, blood sample collected at specimen extraction; D1, blood sample collected on postoperative day 1; D30, blood sample collected on postoperative day 30; NT, no-touch group; C, conventional group; CMC, circulating mesenchymal cell.

A progressive decline in total CCs and CMCs after surgery was observed in both intervention groups but was only significant in the C group (Figure 3). This decrease was mainly due to the significant decrease in CMCs at D1 (from 4.78 to 1.64 CMCs/7.5 mL blood; *p* = 0.035), as no differences were found between S0 and S1 or between D1 and D30 (Tables S1, S2 and S3.2). In all the intervention groups, CMC clusters disappeared at D1 and reappeared at D30.

Table 4 shows the CC counts and variation between the intervention groups and time points.

No significant differences were found between the intervention groups in the absolute and relative CTC and CMC variations between S0 and the remaining time points, suggesting that there is no reduction in CTC or CMC release with NT LRN (Tables S1 and S2). However, a significantly lower CMC count was found in the NT arm compared to the C arm at S1 (4.38 vs. 7.06, *p* = 0.044). There were no differences in CC counts at any other time point.

In the entire RCC cohort, moderate negative correlations were found between CT tumor contrast washout and CMCs at S1 (r = −0.503, *p* = 0.008, and r = −0.425, *p* = 0.027, for corticomedullary and late phases, respectively) and CMC clusters at S0 (r = −0.526, *p* = 0.005, and r = −0.442, *p* = 0.021, for corticomedullary and late phases, respectively). Thus, the greater the washout, the fewer the CMCs (Table S6).

The total CC counts at S0 and ∂S1–S0 correlated with the MAP score (*p* = 0.031 and 0.020, respectively) [25]. The same was true for the CMC counts at ∂S1–S0 (*p* = 0.024).

No differences in the CC counts were found according to the TNM stage, histologic type (clear vs. non-clear cell), International Society of Urological Pathology (ISUP) pathologic grade, PADUA score [26], R.E.N.A.L. score [27], and tumor size (Table S5). In addition, no significant correlation was found between the CTC or CMC counts and tumor diameter, volume, parenchymal contact area, or attenuation value measured by a CT scan (Table S6).

At a median follow-up of 12.5 months, no differences in complication rates, PFS, or OS were observed between groups (Figure S3). Subgroup analyses by CC type and count also showed no survival differences.

## 4. Discussion

This study suggests that an NT LRN does not reduce CTC or CMC release or impact survival. It also shows no increase in CC release with surgical manipulation. Similar findings by Haga et al. showed no differences in CTC counts after LRN versus open RN (7.7 ± 6.8 to 22.5 ± 26.3, *p* < 0.05) [28]. The study suggested that minimally invasive surgery reduced CC release. Conversely, two studies reported an increase in CTC detection rate after RCC surgery, but CTC counts were not studied [14].

In the present study, a progressive decrease in CMC counts was observed over time, most of which occurred at D1. This was significant only in the C group, probably due to a randomly higher baseline count and small sample size. Similar findings of a progressive decrease in CTC counts during follow-up in M0 patients were reported by Wang et al. [29].

CTC detection rates in this study were low, possibly due to the predominance of localized, low-stage tumors. Additionally, RCC typically has lower epithelial CTC counts due to a higher incidence of EMT with the loss of epithelial markers compared to other tumor types [5,6]. Although a progressive decrease in CTCs was observed across time points, it was not significant, likely due to low baseline counts and a small sample size.

The only clinicopathologic parameter that correlated with the total CC and CMC counts was the MAP score, an imaging surrogate marker of perirenal inflammation. No other clinicopathologic or laboratory parameters correlated with CC counts, and the same was observed for tumor diameter, volume, parenchymal contact area, or attenuation value.

The NT technique proved to be faster and as safe as the C technique. NT RN took on average 23 min less than the C technique, which is an advantage per se and may favor the choice of this technique. Although several technological advances have helped surgeons reduce operative times, such as the use of advanced bipolar energy and intraoperative imaging, surgical technical optimization, as described in this article, is still critical for optimized outcomes [30].

No CCs were identified in the healthy control group. However, despite the absence of CTCs, a significant number of CMCs were identified in all the patients in the chronic inflammation control group. The same was true for the oncocytoma patients. None of these patients developed cancer during follow-up. Furthermore, the CMC counts in these two groups did not differ from those of the RCC patients. This was surprising because the literature reports the absence of CCs in controls using the same criteria and raises the question of the nature of CMCs [5]. The scientific community has referred to these cells as mesenchymal CTCs. To our knowledge, no study to date has included an inflammation control group. We may wonder whether these cells are CTCs or a subset of leukocytes with low CD45 expression, cancer-associated fibroblasts, or some other inflammatory cell. They may even be different cell types. There is a well-studied relationship between cancer and inflammation that may help explain these findings [31]. The correlation between CMC counts and MAP scores also points in this direction. However, the decrease in CMC counts after surgery suggests that they are mainly of tumor origin. The characterization of CCs with four markers is a limitation of most detection devices [5]. Caution should be exercised in classifying these cells as CTCs. Future downstream CMC analysis and improved biomarker selection are warranted to elucidate their true nature.

The main limitations of this study were its small sample size, low power to detect small differences in CC counts between groups and time points, and short follow-up, which hindered clinical outcome analysis. In addition, the downstream analysis of CC was not performed.

Perioperative CTC kinetics is still poorly understood, and its clinical significance remains unclear. This study does not suggest an advantage of early pedicle ligation. Has the last Robson’s principle fallen? Larger studies are crucial to confirm this hypothesis. In addition, a more comprehensive analysis of CCs and their interaction with the immune system may increase our understanding of RCC and improve future treatment protocols.

## 5. Patents

RUBYchip™ is based on patent PCT/EP2016/078406, filed by INL in front of EPO on 22 November 2016, covering the geometry and surface coating of the microfluidic system for CTC isolation, and currently licensed exclusively to RUBYnanomed.

## 6. Conclusions

NT LRN did not reduce CC release or improve survival compared to C LRN. However, it proved to be faster and as safe as the conventional technique. CMCs were found in patients with chronic inflammation and oncocytoma and decreased after surgery, suggesting tumor origin but questioning their CTC status.

## Figures and Tables

**Table 1:** Clinicopathologic characteristics of the study population according to the intervention groups and study control.

	NT Group n = 16	C Group n = 16	*p*-Value ^†^	Control Group n = 9	*p*-Value ^±^
Age at surgery (yr), median (quartile)	61.5 (56.8–70.5)	61.0 (55.8–71.0)	0.985	65 (55.0–69.0)	0.912
Gender M, n (%)	9 (56.25%)	13 (81.25%)	0.252	5 (80%)	0.693
BMI (kg/m^2^), median (quartile)	26.2 (23.6–30.1)	26.9 (24.1–31.4)	0.509	29.3 (27.0–30.1)	0.637
Smoking, n (%)	6 (37.5%)	4 (25%)	0.704	3 (33.3%)	1
Obesity, n (%)	5 (31.3%)	5 (31.3%)	1	2 (22.2%)	0.702
Hypertension, n (%)	11 (68.8%)	13 (81.3%)	0.685	6 (66.7%)	0.680
Diabetes, n (%)	2 (12.5%)	1 (6.25%)	1	4 (44.4%)	0.031
Antiplatelet therapy, n (%)	5 (31.3%)	5 (31.3%)	1	2 (22.2%)	0.702
Anticoagulation, n (%)	1 (6.25%)	5 (31.3%)	0.172	0	0.309
ECOG, n (%)			0.654		0.028
0	6 (37.5%)	8 (50%)		2 (22.2%)	
1	7 (43.8%)	4 (25%)		5 (55.6%)	
2	3 (18.8%)	4 (25%)		0	
3	0	0		2 (22.2%)	
GFR (mL/min/1.73), median (quartile)	64.5 (38.3–81.25)	81.5 (61.8–97.0)	0.239	73.0 (54.0–90.0)	0.935
CKD history, n (%)			0.273		1
0	8 (50%)	11 (68.8%)		5 (55.6%)	
1	8 (50%)	4 (25%)		4 (44.4%)	
2	0	1 (6.3%)		0	
Dialysis, n (%)	3 (18.8%)	1 (6.3%)	0.600	0	0.559
Autoimmune disease, n (%)	3 (18.8%)	1 (6.3%)	0.600	0	0.559
Tumor characteristics					
cT			1		1
cT1a, n (%)	3 (18.8%)	2 (12.5%)		0	
cT1b, n (%)	5 (31.3%)	6 (37.5%)		0	
cT2a, n (%)	3 (18.8%)	4 (25%)		0	
cT2b, n (%)	2 (12.5%)	1 (6.3%)		0	
cT3a, n (%)	2 (12.5%)	3 (18.8%)		0	
cT3b, n (%)	1 (6.3%)	0		0	
cN			1		1
cN0, n (%)	16 (100%)	15 (93.75%)		0	
cN1, n (%)	0	1 (6.3%)		0	
cM			0.484		1
M0, n (%)	16 (100%)	14 (87.5)		0	
M1, n (%)	0	1 (6.3%)		0	
Mx, n (%)	0	1 (6.3%)		0	
Tumor side			0.716		0.039
Right, n (%)	11 (68.8)	9 (56.3%)		9 (100%)	
Left, n (%)	5 (31.3%)	7 (43.8%)		0	
Tumor size (mm), median (quartile)	59.5 (40.8–98.3)	59.0 (49.0–75.5)	0.865	NA	NA
Perioperative characteristics					
ASA classification, n (%)			0.767		1
I	1 (6.3%)	1 (6.3%)		0	
II	7 (43.8%)	6 (37.5%)		0	
III	8 (50%)	7 (43.8%)		0	
IV	0	2 (12.5%)		0	
Blood loss (mL), median (quartile)	100.0 (50.5–212.5)	90.0 (50.0–375.0)	0.849	NA	NA
Operative time (min), median (quartile)	84.5 (63.8–95.3)	107.5 (96.3–131.8)	0.015 *	NA	NA
Time until renal vein exposure (min), median (quartile)	21.0 (16.5–27.0)	37.0 (28.3–55.0)	0.001 *	NA	NA
Time between renal vein exposure and ligation (min), median (quartile)	6.5 (3.0–13.5)	16.5 (13.0–32.3)	0.002 *	NA	NA
Days of hospital stay (days), median (quartile)	2.5 (2.0–3.3)	3.0 (2.0–4.3)	0.405	NA	NA
Complications, n (%)	3 (18.8%)	3 (18.8%)	1	0	1
Clavien–Dindo I	0	1 (6.3%)		0	
Clavien–Dindo II	2 (12.5%)	1 (6.3%)		0	
Clavien–Dindo Iva	1 (6.3%)	1 (6.3%)		0	
Pathological parameters					
Tumor diameter (mm), median (quartile)	50.0 (40.0–107.5)	64.5 (45.0–70.5)	0.890	NA	NA
Histologic type, n (%)			0.281		1
Clear cell	6	11		0	
Papillary type 1	0	1		0	
Papillary type 2	1	1		0	
Chromophobe	3	0		0	
Other RCC	2	2		0	
Oncocytoma	3	1		0	
Xanthogranulomatous Pyelonephritis	1	0		0	
Pathology grade (Fuhrman), n (%)			0.793		1
1	3	2		0	
2	8	11		0	
3	0	0		0	
4	0	1		0	
Microvascular invasion, n (%)	2	3	1	0	1
Lymphatic invasion, n (%)	1	1	1	0	1
Renal vein (segmental) invasion, n (%)	0	4	0.106	0	1
Collecting system invasion, n (%)	1	1	1	0	1
Perirenal fat invasion, n (%)	3	3	1	0	1
pT, n (%)			0.208		1
pT1a	3	4		0	
pT1b	5	3		0	
pT2a	0	3		0	
pT2b	2	0		0	
pT3a	2	5		0	
pT3b	0	0		0	
pN, n (%)			NA		NA
pN0	4	6		0	
pN1	0	0		0	
Positive surgical margins, n (%)	0	0		NA	

* clinically significant; ^†^ comparison between no-touch and control groups; ^±^ comparison between intervention and control groups. ASA, American Society of Anesthesiologists; BMI, body mass index; C, control group; CKD, chronic kidney disease; ECOG, Eastern Cooperative Oncology Group performance status; GFR, glomerular filtration rate; NT, no-touch; M, male; min, minute; mL, milliliter; n, number; NA, not available; RCC, renal cell carcinoma.

**Table 2:** Baseline (S0) circulating cell counts and characterization in intervention and control groups.

	Control Groups n = 9		Intervention Groups (S0) n = 31									
	HC n = 5	IC n = 4	RCC NT n = 12	RCC C n = 15	Oncocytoma n = 4	IC vs. HC *p* Value	IC vs. RCC C *p* Value	IC vs. RCC NT *p* Value	IC vs. O *p* Value	O vs. HC *p* Value	O vs. RCC C *p* Value	O vs. RCC NT *p* Value
Single CTCs	0	0	0	0.33 (0, 0, 5)	0	1	1	1	1	1	1	1
Epithelial CTCs	0	0	0	0.33 (0, 0, 5)	0	1	1	1	1	1	1	1
EMT CTCs	0	0	0	0	0	NA	NA	NA	NA	NA	NA	NA
CMCs	0	22.5 (1, 18.5, 52)	3.0 (0, 1.0, 12)	8.47 (0, 4.0, 33)	24.75 (8, 17.0, 57)	0.017 *	0.874	0.233	1	0.002 *	0.205	0.037 *
Single CMCs	0	11.75 (1, 12.0, 22)	2.58 (0, 1.0, 11)	7.2 (0, 4.0, 31)	14.25 (8, 15.0, 19)	0.018 *	1	0.227	1	0.003 *	0.371	0.052
CMCs in clusters	0	10.75 (0, 6.5, 30)	0.42 (0, 0, 3)	1.27 (0, 0, 10)	10.5 (0, 2.0, 38)	0.174	0.431	0.314	1	0.213	0.567	0.387
Clusters	0	2 (0, 1.0, 6)	0.17 (0, 0, 1)	0.4 (0, 0, 3)	2 (0, 1.0, 6)	0.202	0.520	0.360	1	0.202	0.520	0.360
Total CCs	0	22.5 (1, 18.5, 52)	3.0 (0, 1.0, 12)	8.8 (0, 5.0, 33)	24.75 (8, 17.0, 57)	0.017 *	1	0.234	1	0.002 *	0.269	0.030 *
Single CCs	0	11.75 (1, 12.0, 22)	2.58 (0, 1.0, 11)	7.53 (0, 5.0, 31)	14.25 (8, 15.0, 19)	0.019 *	1	0.225	1	0.003 *	0.476	0.042 *

Values presented as minimum, median, and maximum; * clinically significant; *p*-values were calculated using the Kruskal–Wallis test for each cell count variable to assess the homogeneity of the four groups, followed by pairwise comparisons (using Dunn’s test) with Bonferroni correction. C, conventional; CC, circulating cell; CMC, circulating mesenchymal cell; CTC, circulating tumor cell; EMT, epithelial-to-mesenchymal transition; HC, healthy control; IC, inflammatory control; n, number; NA, not available; NT, no-touch; RCC, renal cell carcinoma; O, oncocytoma; S0, time of arrival in the operating room (baseline).

**Table 3:** CC counts and characterization by study group and time point.

	RCC NT Group (n = 12)				RCC C Group (n = 15)			
	S0	S1	D1	D30	S0	S1	D1	D30
Single CTCs	0	0	1.36 (0, 0, 13)	0.09 (0, 0, 1)	0.33 (0, 0, 5)	0.07 (0, 0, 1)	0.07 (0, 0, 1)	0.36 (0, 0, 4)
Epithelial CTCs	0	0	0.36 (0, 0, 2)	0.09 (0, 0, 1)	0.33 (0, 0, 5)	0.07 (0, 0, 1)	0.07 (0, 0, 1)	0.36 (0, 0, 4)
EMT CTCs	0	0	1.00 (0, 0, 11)	0	0	0	0	0
CMCs	3.00 (0, 1.00, 5)	2.00 (0, 0, 11)	1.91 (0, 0, 12)	0.91 (0, 0, 7)	8.47 (0, 4.00, 33)	7.00 (0, 4.00, 31)	1.43 (0, 1.00, 6)	1.57 (0, 0.50, 11)
Single CMCs	2.58 (0, 1.00, 11)	2.00 (0, 0, 11)	1.91 (0, 0, 12)	0.45 (0, 0, 2)	7.20 (0, 4.00, 31)	6.27 (0, 4.00, 22)	1.43 (0, 1.00, 6)	1.21 (0, 0.50, 6)
CMCs in clusters	0.42 (0, 0, 3)	0	0	0.45 (0, 0, 5)	1.27 (0, 0, 10)	0.73 (0, 0, 11)	0	0.36 (0, 0, 5)
Clusters	0.17 (0, 0, 1)	0	0	0.18 (0, 0, 2)	0.40 (0, 0, 5)	0.20 (0, 0, 3)	0	0.07 (0, 0, 1)
Total CCs	3.00 (0, 1.00, 12)	2.00 (0, 0, 11)	7.00 (0, 1.00, 25)	1.00 (0, 0, 8)	8.80 (0, 5.00, 33)	7.07 (0, 4.00, 31)	1.5 (0, 1.00, 6)	1.93 (0, 1.00, 11)
Single CCs	2.58 (0, 1.00, 11)	2.00 (0, 0, 11)	3.27 (0, 1.00, 25)	0.55 (0, 0, 2)	7.53 (0, 5.00, 31)	6.33 (0, 4.00, 22)	1.5 (0, 1.00, 6)	1.57 (0, 1.00, 6)
	**Oncocytoma (n = 4)**				**Xanthogranulomatous pyelonephritis (n = 1)**			
	**S0**	**S1**	**D1**	**D30**	**S0**	**S1**	**D1**	**D30**
Single CTCs	0	0	0	0	0	0	0	0
Epithelial CTCs	0	0	0	0	0	0	0	0
EMT CTCs	0	0	0	0	0	0	0	0
CMCs	24.75 (8, 17.00, 57)	13.5 (1, 11.00, 31)	0.75 (0, 0.50, 2)	5.50 (0, 6.00, 12)	4	0	1	1
Single CMCs	14.25 (8, 15.00, 19)	6.25 (1, 6.00, 12)	0.75 (0, 0.50, 2)	4.75 (0, 5.00, 9)	4	0	1	1
CMCs in clusters	10.50 (0, 2.00, 38)	7.25 (0, 1.00, 27)	0	0.75 (0, 0, 3)	0	0	0	0
Clusters	2.00 (0, 1.00, 6)	0.50 (0, 0.5, 1)	0	0.25 (0, 0, 1)	0	0	0	0
Total CCs	24.75 (8, 17.00, 57)	13.50 (1, 11.00, 31)	0.75 (0, 0.50, 2)	5.50 (0, 6.00, 10)	4	0	1	1
Single CCs	14.25 (8, 15.00, 19)	6.25 (1, 6.00, 12)	0.75 (0, 0.50, 2)	4.75 (0, 5.00, 9)	4	0	1	1

Values presented as minimum, median, and maximum; S0, time of arrival in the operating room (baseline); S1, time of specimen extraction; D1, postoperative day 1; D30, postoperative day 30; C, conventional; CC, circulating cell; CMC, circulating mesenchymal cell; CTC, circulating tumor cell; EMT, epithelial-to-mesenchymal transition; NT, no-touch; RCC, renal cell carcinoma.

**Table 4:** CTC counts and variation in each intervention group and time point (primary outcome).

	S0			S1			D1			D30			Cell Count Difference S1–S0			Relative Cell Count Difference S1–S0 (%)			Cell Count Difference D1–S0			Relative Cell Count Difference D1–S0 (%)		
	NT	C	*p*-Value *	NT	C	*p*-Value *	NT	C	*p*-Value *	NT	C	*p*-Value *	NT	C	*p*-Value ^±^	NT	C	*p*-Value ^±^	NT	C	*p*-Value ^±^	NT	C	*p*-Value ^±^
Single CTCs	0	0.33	0.371	0	0.07	0.371	1.36	0.07	0.169	0.09	0.36	0.662	0	−0.26	0.412	NA	−80	NA	1.36	−0.26	0.126	NA	−78.6	NA
Epithelial CTCs	0	0.33	0.371	0	0.07	0.371	0.36	0.07	0.169	0.09	0.36	0.662	0	−0.26	0.412	NA	−80	NA	0.36	−0.26	0.126	NA	−78.6	NA
EMT CTCs	0	0	NA	0	0	NA	1	0	0.259	0	0	NA	0	0	NA	NA	NA	NA	1	0	0.295	NA	NA	NA
CMCs	3.00	8.47	0.251	2.00	7.00	0.033	1.91	1.43	0.554	0.91	1.57	0.302	−1.00	−1.47	0.883	−33.3	−17.3	0.268	−1.09	−7.04	0.754	−36.36	−83.1	0.311
Single CMCs	2.58	7.20	0.203	2.00	6.27	0.033	1.91	1.43	0.554	0.45	1.21	0.235	−0.58	−0.93	0.677	−22.6	−13.0	0.268	−0.67	−5.77	0.213	−100.0	−100.0	0.311
CMCs in clusters	0.42	1.27	0.746	0.00	0.73	0.371	0.00	0.00	NA	0.45	0.357	0.861	−0.42	−0.54	1	−100.0	−42.1	NA	−0.42	−1.27	0.784	−100.0	−100.0	NA
Clusters	0.17	0.40	0.719	0.00	0.20	0.371	0.00	0.00	NA	0.18	0.07	0.846	−0.17	−0.20	1	−100.0	−50.0	NA	−0.17	−0.40	0.213	−100.0	−100.0	NA
Total CCs	3.00	8.80	0.142	2.00	7.07	0.021	7.00	1.50	0.775	1.00	1.93	0.373	−1.00	−1.73	0.961	−33.3	−19.7	0.283	4.00	−7.30	0.077	9.1	−83.0	0.644
Single CCs	2.58	7.53	0.106	2.00	6.33	0.021	3.27	1.50	0.775	0.55	1.57	0.270	−0.58	−1.20	0.826	−22.6	−15.9	0.283	0.69	−6.03	0.077	26.7	−80.1	0.607

Values presented as means; * Kruskal–Wallis test; ***^±^*** Wilcoxon test. S0, blood sample collected on arrival in the operating room; S1, blood sample collected at specimen extraction; D1, blood sample collected on postoperative day 1; D30, blood sample collected on postoperative day 30; NT, no-touch group; C, conventional group; CC, circulating cell; EMT, epithelial-to-mesenchymal transition; CMC, circulating mesenchymal cell; CTC, circulating tumor cell; NA, not available.

## Data Availability

The data generated during and/or analyzed during the current study are available from the corresponding author upon reasonable request.

## Supplementary Materials

The following supporting information can be downloaded at https://www.mdpi.com/article/10.3390/cancers16213601/s1, Table S1: CC count time point delta differences between RCC intervention groups; Table S2: CC count relative time point delta differences between RCC intervention groups and negativation rates; Table S3.1: CC counts for the whole intervention group according to time point; Table S3.2: CC count differences between time points in the whole intervention group; Table S3.3: CC count differences between clear cell and non-clear cell RCC; Table S3.4: CC count differences between RCC intervention groups according to time between renal vein exposure and ligation; Table S4: Correlation between CC counts and clinicopathological variables according to group; Table S5: Correlation between CC counts and clinicopathological variables at S0 and S1–S0 delta; Table S6: Correlation coefficients for the relation between CC counts and CT imaging variables; Figure S1: Study protocol flow chart; Figure S2: CMC counts per patient and time points; Figure S3: Total CMCs in NT and C groups across timepoints; Figure S4 Kaplan–Meyer survival analysis; Figure S5: CONSORT 2010 statement flow diagram.
