# Supplementary figures and images for: A Randomized Controlled Trial Assessing the Release of Circulating Tumor and Mesenchymal Cells in No-Touch Radical Nephrectomy
Source: Cancers (Basel). 2024 Oct 25;16(21):3601. doi: 10.3390/cancers16213601 (PMC11545310; doi:10.3390/cancers16213601)

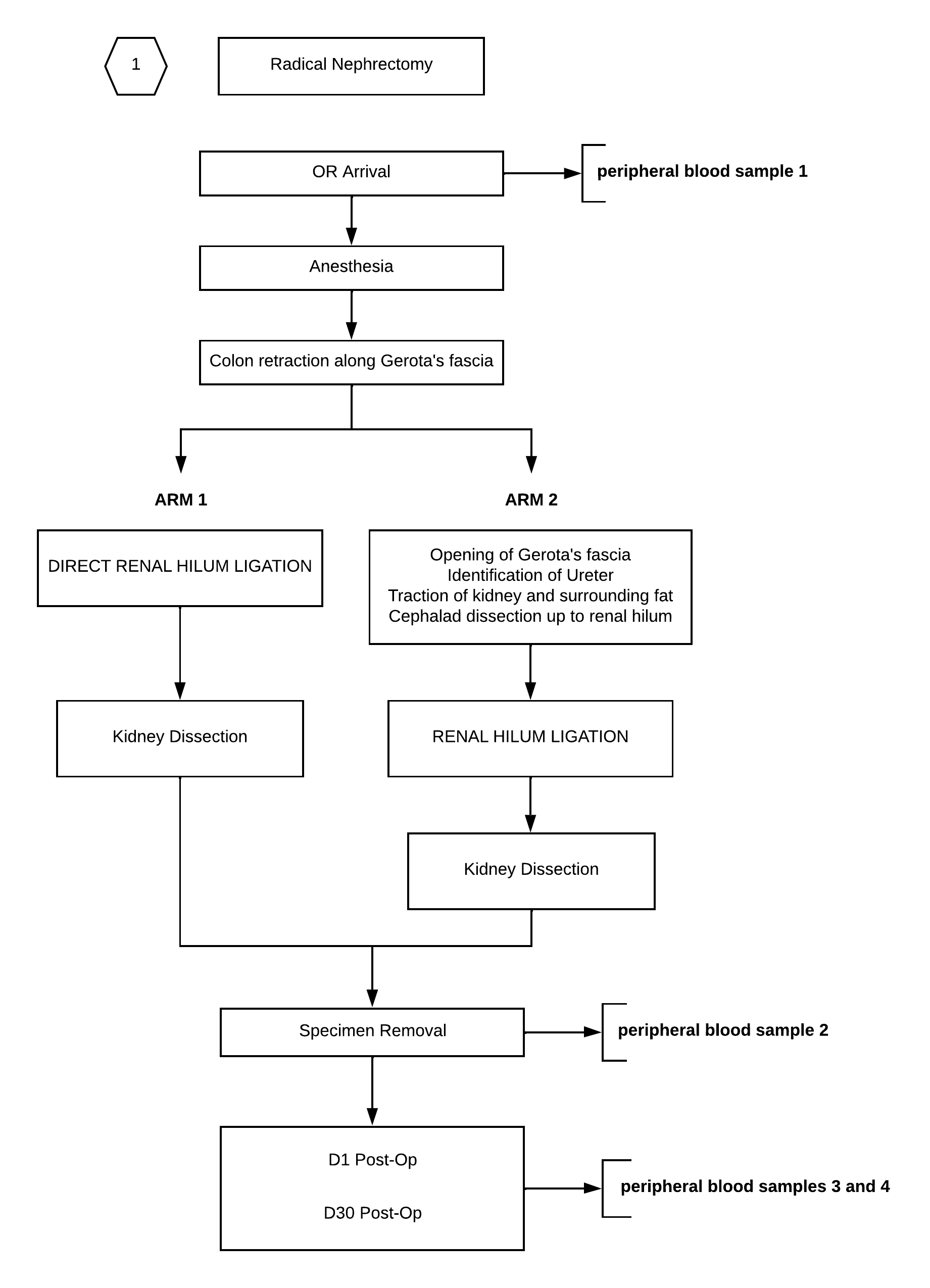

Supplement: Supplementary file 1 [file cancers-16-03601-s001.zip › Figure S1.png]

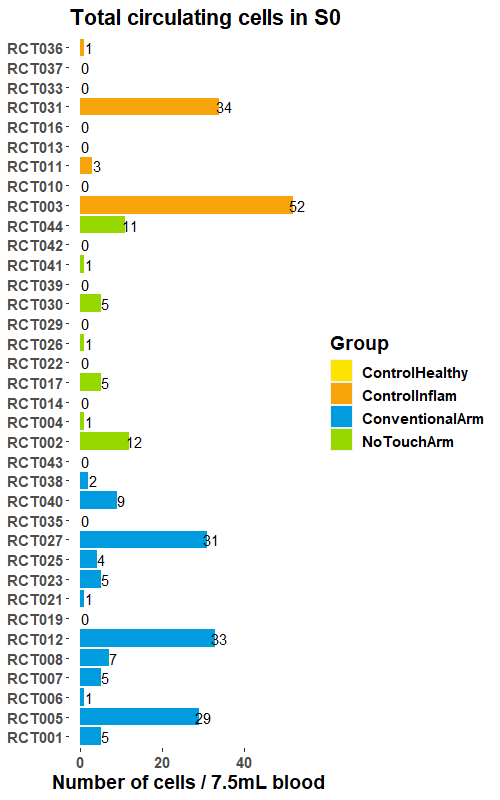

Supplement: Supplementary file 1 [file cancers-16-03601-s001.zip › Figure S2.png]

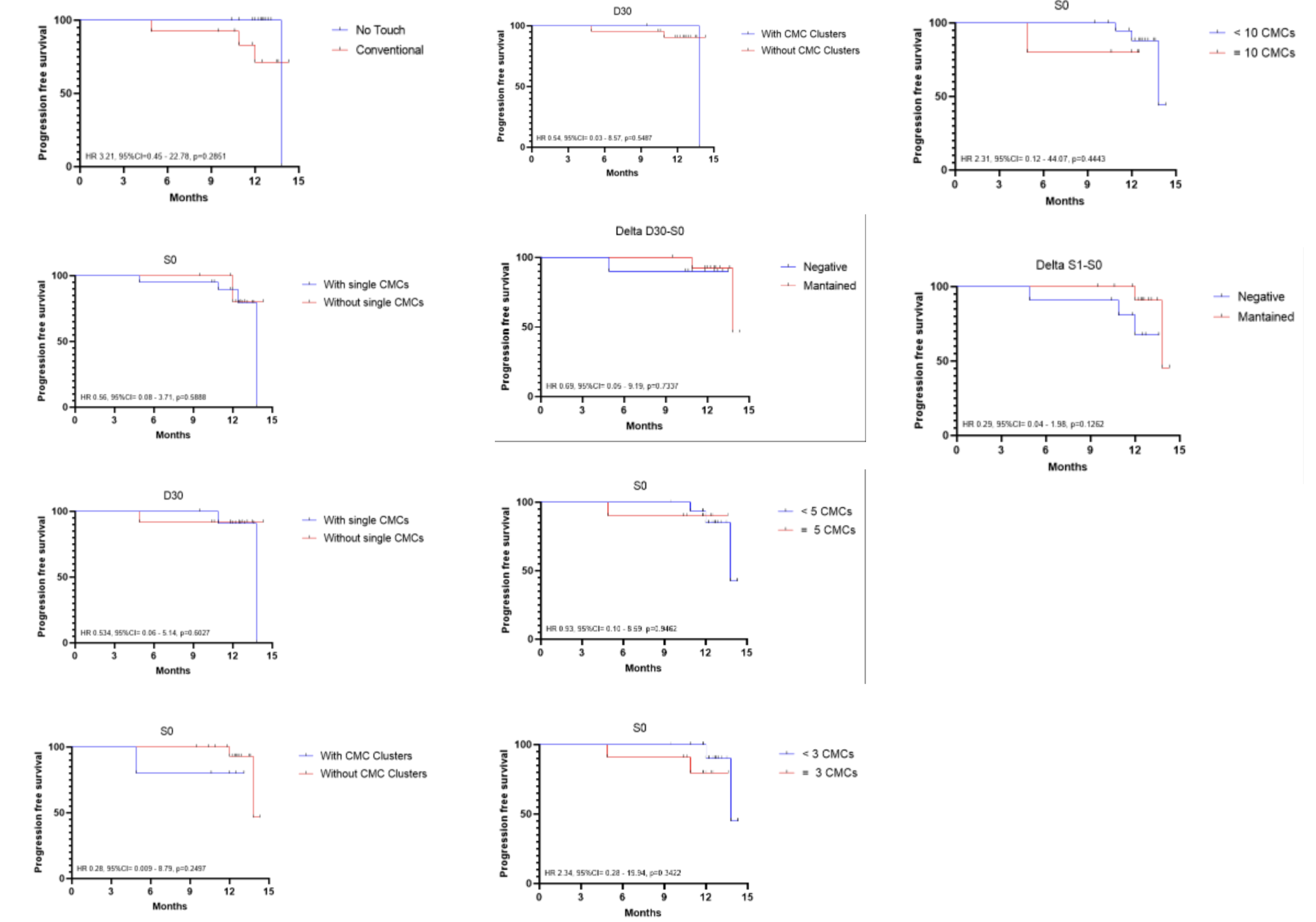

Supplement: Supplementary file 1 [file cancers-16-03601-s001.zip › Figure S3.png]

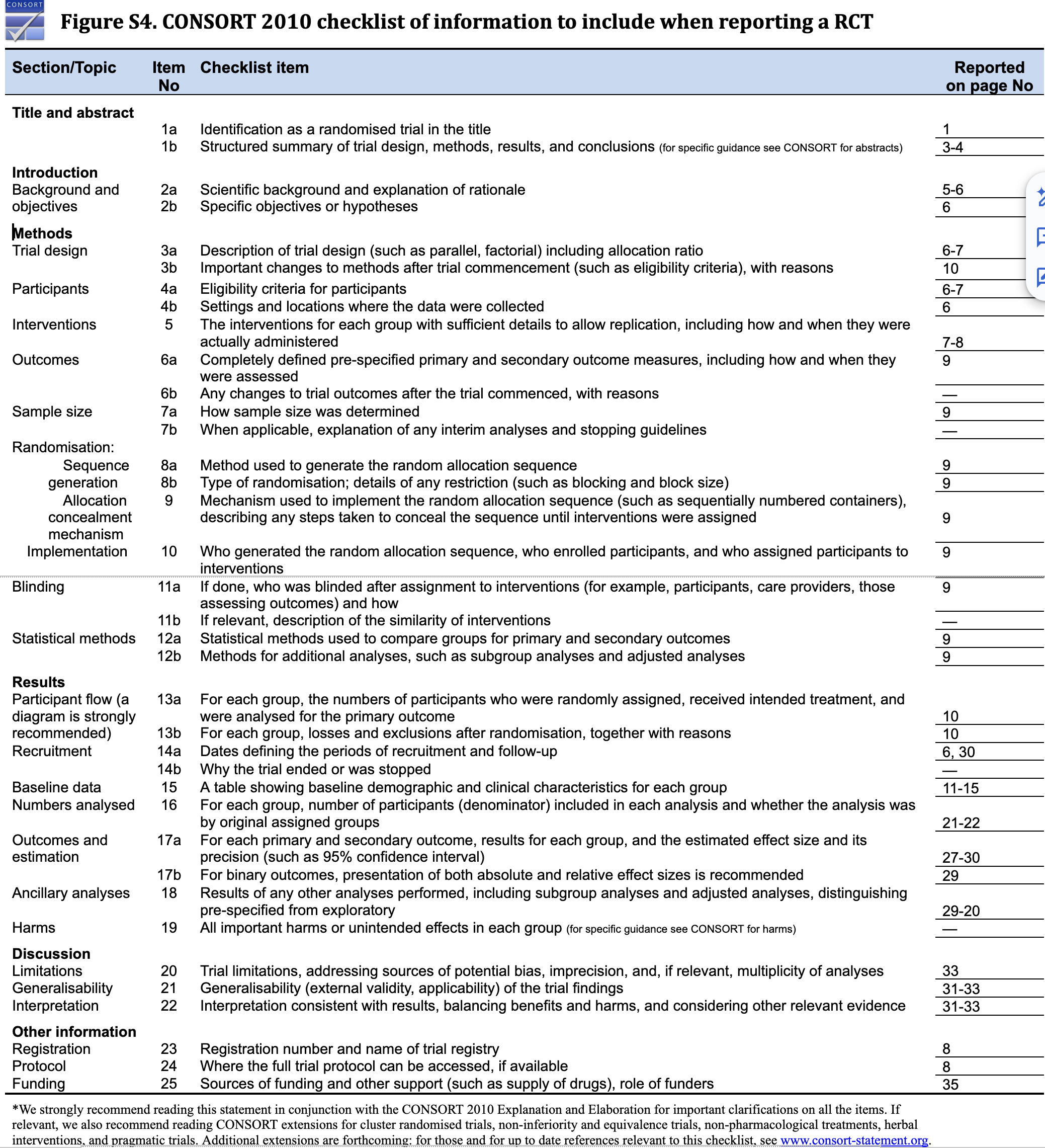

Supplement: Supplementary file 1 [file cancers-16-03601-s001.zip › Figure S4.png]

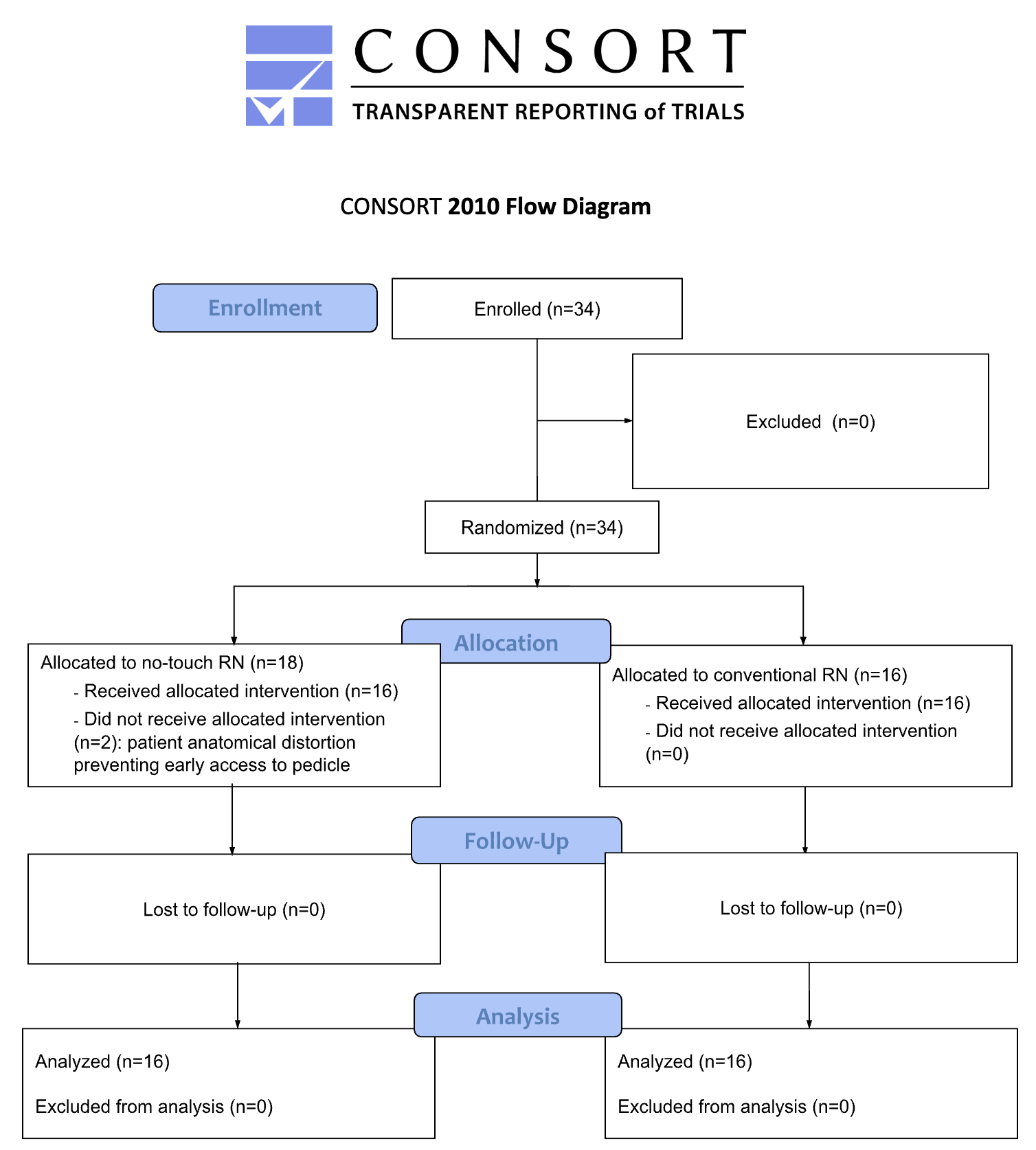

Supplement: Supplementary file 1 [file cancers-16-03601-s001.zip › Figure S5.png]
